# McDowell Medical Society

**Published:** 1880-05-20

**Authors:** 


### *McDOWELL MEDICAL SOCIETY, HENDERSON, KY.*

Dr. J. T. Jenkins, the Corresponding Secretary, gives notice that the approaching session will be one of great interest, as matters of great importance will be brought before the meeting. A number of prominent physicians will be present. All members having papers to read (both voluntary and reports of committees) are urgently requested to notify the Chairman of the Committee of Arrangements, Dr. W. B. Furman, by the fifteenth of May.

Every preparation will be made by the Committee of Arrangements for the comfort and pleasure of those attending, and it is earnestly requested that all members who can possibly do so will be present.

The Session will open on May 26, 1880.

